# Higher Cholesterol Absorption Marker at Baseline Predicts Fewer Cardiovascular Events in Elderly Patients Receiving Hypercholesterolemia Treatment: The KEEP Study

**DOI:** 10.1161/JAHA.123.031865

**Published:** 2024-01-19

**Authors:** Masanari Kuwabara, Jun Sasaki, Yasuyoshi Ouchi, Shinichi Oikawa, Kiyotaka Nakagawa, Masao Sato, Shinji Koba, Suminori Kono, Tetsunori Saikawa, Hidenori Arai

**Affiliations:** ^1^ Toranomon Hospital Tokyo Japan; ^2^ International University of Health and Welfare Fukuoka Japan; ^3^ Fukujuji Hospital Tokyo Japan; ^4^ Tohoku University Miyagi Japan; ^5^ Kyushu University Fukuoka Japan; ^6^ Showa University Tokyo Japan; ^7^ MedStat Corporation Fukuoka Japan; ^8^ Oita San‐Ai Medical Center Oita Japan; ^9^ National Center for Geriatrics and Gerontology Aichi Japan

**Keywords:** atherosclerotic cardiovascular disease, cholesterol absorption marker, elderly, ezetimibe, primary prevention, Cardiovascular Disease, Primary Prevention, Pharmacology

## Abstract

**Background:**

Higher cholesterol absorption has been reported to be related to a higher incidence of cardiovascular events (CVEs). The KEEP (Kyushu Elderly Ezetimibe Phytosterol) study, a substudy of the EWTOPIA 75 (Ezetimibe Lipid‐Lowering Trial on Prevention of Atherosclerotic Cardiovascular Disease in 75 or Older) study, investigated the relationships of cholesterol absorption and synthesis markers with CVEs in older old individuals with hypercholesterolemia, particularly in relation to ezetimibe treatment.

**Methods and Results:**

Eligible patients were those aged ≥75 years who had low‐density lipoprotein cholesterol ≥140 mg/dL, no history of coronary artery disease, and no recent use of lipid‐lowering drugs. Participants were randomly assigned into a diet‐only or diet‐plus‐ezetimibe group. Baseline and 24‐week follow‐up blood samples were analyzed for cholesterol absorption (eg, campesterol) and synthesis markers (eg, lathosterol). Of 1287 patients, 1061 patients with baseline measurement were analyzed. Over a median follow‐up of 4.0 years, 64 CVEs occurred. Higher campesterol levels at baseline were significantly associated with a lower risk of CVEs. After adjustment for sex, age, and treatment, the hazard ratios for the lowest to highest quartile categories of baseline campesterol were 1.00 (reference), 0.59 (95% CI, 0.30–1.17), 0.44 (95% CI, 0.21–0.94), and 0.44 (95% CI, 0.21–0.93), respectively (trend *P*=0.01). This association persisted after further adjustment for hypertension, diabetes, and other cardiovascular risk factors. Neither interactions with ezetimibe treatment nor mediating effects of the changes in cholesterol absorption markers were observed.

**Conclusions:**

The KEEP study indicated that higher campesterol levels without lipid‐lowering drugs were associated with a lower incidence of CVEs in older old individuals with hypercholesterolemia who were subsequently treated with diet or ezetimibe.

**Registration:**

URL: https://www.umin.ac.jp; unique identifier: UMIN000017769.

**Nonstandard Abbreviations and Acronyms**
CVE: cardiovascular event
EPIC‐Norfolk: European Prospective Investigation Into Cancer–Norfolk
EWTOPIA 75: Ezetimibe Lipid‐Lowering Trial on Prevention of Atherosclerotic Cardiovascular Disease in 75 or Older
HIJ‐PROPER: Heart Institute of Japan Proper Level of Lipid Lowering With Pitavastatin and Ezetimibe in Acute Coronary Syndrome
IC: informed consent
KEEP: Kyushu Elderly Ezetimibe Phytosterol
MEGA: Management of Elevated Cholesterol in the Primary Prevention Group of Adult Japanese
PCOOH: phosphatidylcholine hydroperoxide

**Clinical Perspective**

**What Is New?**

- The KEEP (Kyushu Elderly Ezetimibe Phytosterol) study investigated the relationships of cholesterol absorption and synthesis markers with cardiovascular events.
- Higher levels of campesterol, a cholesterol absorption marker, without lipid‐lowering drugs were associated with a lower incidence of cardiovascular events in older old individuals with hypercholesterolemia who were subsequently treated with diet or ezetimibe.

**What Are the Clinical Implications?**

- Further research is needed to confirm these findings in other populations and to investigate the mechanisms underlying the association between cholesterol absorption markers and cardiovascular events.

Phytosterols have drawn much attention in the management of hypercholesterolemia and prevention of cardiovascular disease, not only because of potential atherogenicity of plant sterols^1^ but also due to clinical utility as cholesterol metabolism markers.^2^ Serum contains small amounts of noncholesterol sterols/stanols, which are used as cholesterol synthesis and absorption markers.^3^, ^4^ Cholesterol precursors such as lathosterol and desmosterol are synthesis markers, and plant sterols (eg, campesterol and sitosterol) and cholestanol are absorption markers. Cholestanol is a metabolite of cholesterol, but serum cholestanol levels are closely correlated negatively with cholesterol synthesis and positively with cholesterol absorption.^5^ Statins inhibit cholesterol synthesis and enhance cholesterol absorption.^6^, ^7^ Ezetimibe decreases serum low‐density lipoprotein cholesterol (LDL‐C) levels via inhibition of cholesterol absorption but increases cholesterol synthesis.^8^, ^9^ Cholesterol absorption and synthesis may modify the effect of cholesterol‐lowering drugs on cardiovascular disease risk.^10^, ^11^ In the Scandinavian Simvastatin Survival Study of patients with coronary artery disease, the effect of simvastatin on reducing the recurrence events was nullified among patients at the highest quartile of baseline serum cholestanol.^10^ In a subanalysis of the HIJ‐PROPER (Heart Institute of Japan Proper Level of Lipid Lowering With Pitavastatin and Ezetimibe in Acute Coronary Syndrome), the effect of ezetimibe on lowering the risk of adverse end points was substantiated among patients with high levels of serum sitosterol.^11^ These findings have led to the idea of individualizing cholesterol‐lowering therapy on the basis of cholesterol absorption and synthesis markers.^1^

We addressed this issue in the KEEP (Kyushu Elderly Ezetimibe Phytosterol) study, a substudy of the EWTOPIA 75 (Ezetimibe Lipid‐Lowering Trial on Prevention of Atherosclerotic Cardiovascular Disease in 75 or Older).^12^ The EWTOPIA 75 study showed that cholesterol‐lowering therapy with ezetimibe reduced the risk of cardiovascular events (CVEs) in older old individuals aged ≥75 years.^12^ The KEEP study measured cholesterol absorption and synthesis markers at baseline and at 24 weeks of treatment.^13^ The present study examined the effects of diet or ezetimibe treatment on cholesterol absorption and synthesis markers and aimed to evaluate the relationships of cholesterol absorption and synthesis markers with CVEs with special reference to their interactions with ezetimibe treatment and the mediating effects of the changes in cholesterol absorption markers in the ezetimibe effect on risk of CVEs.

## Methods

### Trial Design and Participants

The trial design and participant details of the EWTOPIA 75 study have been published.^12^ The KEEP study is a substudy of the EWTOPIA 75 study, a multicenter, prospective, randomized, open‐label, blinded end point trial conducted in Japan, and the subjects in the KEEP study were part of the participants in the EWTOPIA75 study. The KEEP study required an additional informed consent (IC) regarding blood sample collection. The patients included in this analysis were part of the intention‐to‐treat population in the EWTOPIA 75 study^12^ and had their laboratory parameters measured at baseline (Figure). The KEEP study aimed to examine the relationships of cholesterol absorption and synthesis markers and inflammatory and cardiac markers (baseline and posttreatment) with CVEs.

**Figure .**
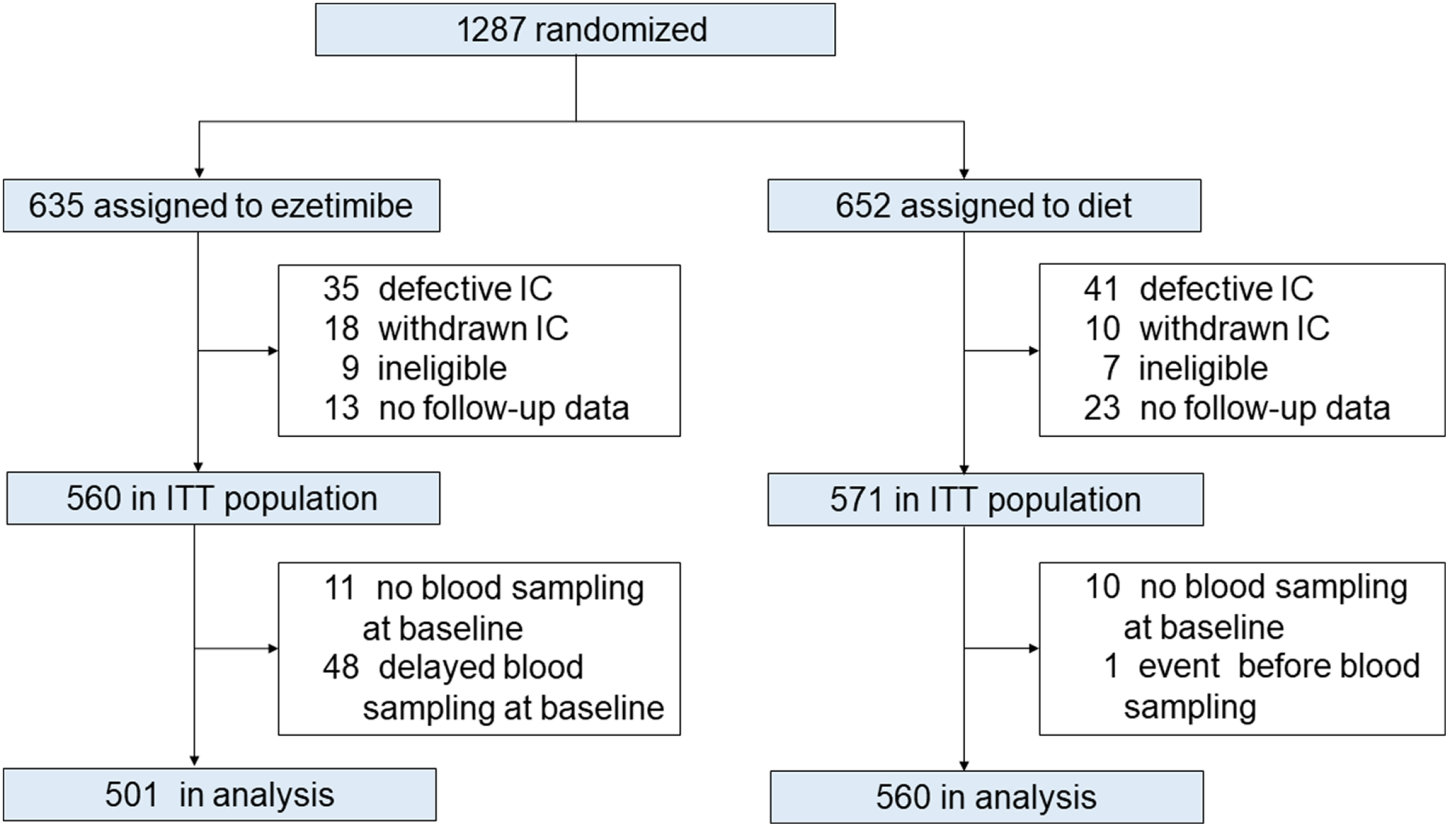
Trial profile. IC indicates informed consent; and ITT, intention‐to‐treat.

The trial enrolled men and women aged ≥75 years without a history of coronary artery disease between May 2, 2009, and December 29, 2014. The inclusion criteria were as follows: acquisition of written IC for participation; age ≥75 years at the time of enrollment; capability to visit the participating site on an ambulatory basis; serum LDL‐C level ≥140 mg/dL as estimated by the Friedewald formula^14^; no use of a lipid‐lowering drug for ≥4 weeks (≥8 weeks in the case of probucol) before the measurement of the baseline serum LDL‐C level; and any of the following complications: diabetes, hypertension, low high‐density lipoprotein cholesterol (HDL‐C), hypertriglyceridemia, current smoking, a history of symptomatic and image‐confirmed stroke, and peripheral artery disease. Key exclusion criteria were as follows: a fasting serum triglyceride level of ≥400 mg/dL; a history of myocardial infarction; a history of coronary revascularization (percutaneous coronary intervention or coronary artery bypass grafting), angina pectoris requiring treatment, stroke within 24 weeks of enrollment, severe liver disease with any 1 of the following 3 items: aspartate aminotransferase (AST) level of ≥100 IU/L, alanine aminotransferase (ALT) level of ≥100 IU/L, or diagnosed cirrhosis; serum creatinine ≥3.0 mg/dL; malignancy; dementia; diagnosis of familial hypercholesterolemia; atrial fibrillation; a history of hypersensitivity to ezetimibe; participation in another clinical trial at the time of enrollment; and inappropriateness for enrollment as judged by the attending physician (eg, hypothyroidism under treatment and nephrotic syndrome). The details of the EWTOPIA 75 study have been published previously.^12^

The trial protocol for the KEEP study was approved by the International University of Health and Welfare and the institutional review board of each participating site. All patients provided written IC before enrollment. This study was conducted in accordance with the principles of the Declaration of Helsinki. The data supporting the findings of this study are available from the corresponding author upon reasonable request.

### Procedures

After obtaining written IC for participation, the patients were randomly assigned to 2 groups: diet therapy plus ezetimibe (10 mg, once daily) in the ezetimibe group and diet therapy alone in the control group. The diet therapy was based on the guidelines of the Japan Atherosclerosis Society, which recommends to decrease the consumption of foods rich in saturated fat and increase the consumption of n‐3 fatty acids, soy foods, vegetables, and fruits, among others.^15^ The follow‐up closed on March 31, 2016.

The KEEP study assessed the following parameters before treatment and 24 weeks after treatment: (1) cholesterol absorption markers including campesterol, cholestanol, 8‐dehydrocholesterol, 7‐dehydrocholesterol, β‐sitosterol, and β‐sitostanol; (2) cholesterol synthesis markers including lathosterol, squalene, and desmosterol; (3) inflammatory markers, hs‐CRP (high‐sensitivity C‐reactive protein); (4) lipoproteins: LDL‐C (direct method), serum total cholesterol, HDL‐C, triglyceride, remnant‐like lipoprotein particle cholesterol, small dense LDL‐C, oxidized phospholipids, oxidized cholesterol, apolipoprotein AI, and apolipoprotein B; (5) cardiac marker: NT‐proBNP (N‐terminal pro‐B‐type natriuretic peptide); and (6) safety: ALT, AST, serum creatinine, and creatinine phosphokinase.

The median follow‐up periods for survivors to death, loss to follow‐up, and March 31, 2016, were equivalent between the 2 trial groups.^12^

### Laboratory Measurements

The study measured various lipid‐related compounds and markers, as well as other blood biomarkers at baseline and 24 weeks of follow‐up. The lipid‐related measurements included total cholesterol, LDL‐C, HDL‐C, triglyceride, apolipoprotein A1 and B, remnant‐like lipoprotein particle cholesterol, small dense LDL‐C, phosphatidylcholine hydroperoxide (PCOOH), phosphatidylcholine, oxycholesterols, and cholesterol absorption and synthesis markers. PCOOH is an oxidized form of phosphatidylcholine and represents oxidized phospholipids. Oxidized phospholipids and oxycholesterols have recently drawn research interest in the development of atherosclerosis and other diseases.^16^, ^17^

The method of measurement for PCOOH and phosphatidylcholine involved the extraction of plasma total lipids with chloroform: methanol (2:1) and purification by solid‐phase extraction to obtain the phospholipid fraction.^18^ The purified phospholipid fraction was dissolved in methanol (200 μL) and used for PCOOH and phosphatidylcholine analyses. PCOOH was detected by chemiluminescence–high‐performance liquid chromatography^19^ and phosphatidylcholine was simultaneously monitored using UV light (210 nm). The concentrations of PCOOH and phosphatidylcholine were calculated using external standard curves. Synthesized PCOOH^20^ and soybean phosphatidylcholine were used as standards.

Other blood biomarkers measured included serum NT‐proBNP, hs‐CRP, total bilirubin, ALT, AST, creatinine, and creatinine phosphokinase levels. The estimated glomerular filtration rate was calculated using serum creatinine concentrations.^21^ Values of hs‐CRP ≥10 mg/L were neglected because of suspected acute inflammation.^22^

Cholesterol absorption and synthesis markers in the extracted serum lipids from 250 μL serum were measured using the method described elsewhere.^23^ The oxycholesterol levels in the serum were analyzed according to the method described previously^24^ with a slight modification regarding the type and amount of the sample and the quantity of the internal standard. In the present study, a serum sample of 250 μL was used with an addition of 100 ng of 5‐cholesten‐3β,19‐diol (19‐hydroxycholesterol) as an internal standard, whereas in the previous study, a lymph sample of 100 μL was used with the addition of 500 ng of the standard substance.

Baseline blood sampling was performed during the period between the date of registration and the start of treatment, and 24‐week blood sampling was performed with allowance of a 4‐week gap. Venous blood samples were drawn after overnight fasting. Serum was separated after centrifugation at the SRL laboratory (Hachioji, Tokyo, Japan) and transported to 3 laboratories for the purpose of the substance to be measured: Tohoku University, Showa University, and Kyushu University.

The number of assayed absorption and synthesis markers was 10 (Figure S1), and the number of analyzed oxycholesterols was 14 (Figure S2). The present study used individual oxycholesterols of the 4 highest concentrations in addition to total oxycholesterols and cholesterol absorption/synthesis markers of the 5 highest concentrations.

Adverse events in laboratory measurements were defined on the basis of the baseline and 24‐week measurements in accordance with the Common Terminology Criteria Adverse Events version 5.0.^25^

### Outcomes

The primary outcome of CVEs in the present analysis was the composite cardiovascular outcome defined in the EWTOPIA 75 study, which was a composite of sudden cardiac death, fatal or nonfatal myocardial infarction, coronary revascularization, or fatal or nonfatal stroke. In cases in which multiple cardiovascular events occurred during the observation period, the first event was counted as a CVE.

### Statistical Analysis

Baseline characteristics of participants were presented as mean±SD, median with interquartile range (the first and third quartiles), or proportions. The median was used for continuous variables with highly skewed distributions. Univariate between‐group comparisons were done by unpaired *t* test, Wilcoxson rank‐sum test, or chi‐square test. The Cox hazard model was used to assess the associations between parameters of interest and primary cardiovascular outcomes. The observation period began with the date of baseline blood sampling and ended with the date of the event, death, loss to follow‐up, or March 31, 2016 (alive without an event). Hazard ratios (HRs) and 95% CIs were calculated according to quartile categories of the baseline parameters, which were created by the *xtile* command in Stata (StataCorp, College Station, TX). Two models were used in statistical adjustment. In model 1, adjustment was made for sex, age (75–79, 80–84, and ≥85 years), and treatment (diet or ezetimibe). In model 2, further adjustment was made for hypertension, diabetes, peripheral artery disease, history of cerebral infarction, prior use of drugs for dyslipidemia, baseline LDL‐C and HDL‐C (quartile categories), and smoking (never, past, and current). The proportional hazards assumption was tested using the Schoenfeld residuals. The assumption was not measurably violated in any of the models used in the present analysis. The effects of the interactions between the treatment group and lipid‐related parameters on the risk of CVEs were assessed using a likelihood ratio test.

The changes from baseline in laboratory measurements at 24 weeks were assessed by analysis of covariance with adjustment for the baseline value. The baseline and follow‐up measurements were transformed to the natural‐logarithm scale for the variables showing a skewed distribution, and mean percentage changes were calculated by antilog transformation of the adjusted means. Regarding the variables without log‐transformation, percentage changes were used in the analysis of covariance. A causal mediation analysis was employed to evaluate the mediating effects of the changes in cholesterol absorption markers in the ezetimibe effect on risk of CVEs.^26^, ^27^ We used the *med4way* command in Stata,^27^ specifying a Cox hazard model for the outcome and a linear regression for the mediator. Adjustment was made for baseline value (quartile categories) of the marker of interest and the covariates specified in the above‐mentioned model 2. Baseline and 24‐week measurements of cholesterol absorption markers were transformed to the natural logarithm scale, and the changes in the log‐transformed values were used as mediator. The 4‐way decomposition was computed with the mediator set at the mean value. The total effect of ezetimibe in terms of excess relative risk was decomposed into 4 components, that is, controlled direct effect, reference interaction, mediated interaction, and pure indirect (mediator) effect. Interpretation of these components has been described in the literatures.^26^, ^27^

The level of statistical significance was set at *P*<0.05 (2‐sided). All statistical analyses were performed using the Stata release 13 software (StataCorp).

### Role of the Funding Source

The KEEP study was funded by the Comprehensive Support Project for Bayer Yakuhin Ltd. and the Okinaka Memorial Institute for Medical Research. The company distributing the trial drug (Bayer Yakuhin, Ltd.) financed the support for the projects. None of these entities played a role in trial design, data analysis, data interpretation, or writing of the manuscript. Y.O. and J.S. had full access to all the data and were responsible for the decision to submit the manuscript for publication.

## Results

### Trial Profile

Between May 2, 2009, and December 29, 2014, 1287 patients were randomly assigned to either of the 2 groups after providing written IC: the ezetimibe group (n=635) receiving diet therapy plus ezetimibe treatment and the control group (n=652) receiving diet therapy alone. Of these, 156 patients were excluded in the analysis for the following reasons: 76 patients had defective IC, 28 had withdrawn IC, 16 were ineligible, and 36 had no follow‐up data. The remaining 1131 patients were the intention‐to‐treat population as defined in the EWTOPIA 75 study. Finally, the analysis included 501 in the ezetimibe group and 560 in the diet group having appropriate baseline blood samples (Figure). In the ezetimibe group, there were 143 patients whose baseline blood sampling occurred within <5 days of ezetimibe treatment. The cutoff point of 5 days was determined on the basis of the observation that LDL‐C level did not change within this period in the present study.

Table 1 presents the baseline characteristics of the study participants. Table 2 shows the levels of cholesterol absorption and synthesis markers, oxycholesterols, and phospholipids at baseline. A higher attrition in the number of patients for phospholipid measurement was observed due to storage damage resulting from the earthquake that primarily affected the northern part of Japan in 2011. Cholesterol absorption and synthesis markers were corrected with total cholesterol, and the correlation was generally weak with LDL‐C, but less so with HDL‐C. Correlation coefficients with LDL‐C were cholestanol −0.12, desmosterol −0.05, lathosterol −0.06, campesterol 0.02, and β‐sitosterol −0.04. Correlation coefficients with HDL‐C were cholestanol −0.01, desmosterol −0.10, lathosterol −0.13, campesterol 0.18, and β‐sitosterol 0.17.

**Table 1:** Baseline Characteristics of the Study Participants

Variable	Both (n=1061)*	Ezetimibe (n=501)	Control (n=560)
Age, y	80.5±4·8	80.5±4.8	80.4 (4.9)
Age category, n (%)			
75–79	523 (49.3)	245 (48.9)	278 (49.6)
80–84	344 (32.4)	162 (32.3)	182 (32.5)
≥85	194 (18.3)	94 (18.8)	100 (17.9)
Sex, n (%)			
Male	253 (23.8)	119 (23.8)	134 (23.9)
Female	808 (76.2)	382 (76.2)	426 (76.1)
Smoking, n (%)			
Never	930 (87.7)	437 (87.2)	493 (88.0)
Past	93 (8.8)	46 (9.2)	47 (8.4)
Current	38 (3.6)	18 (3.6)	20 (3.6)
Body mass index, kg/m^2^	23.4±3.8	23.4±3.5	23.4±4.0
Waist circumference, cm	85.7±9.8	85.7±9.6	85.7±9.9
Systolic blood pressure, mm Hg	135±16	136±16	134±15
Diastolic blood pressure, mm Hg	74±10	74±11	73±10
Total cholesterol, mg/dL	236±33	236±33	236±33
Non–HDL‐C, mg/dL	180±31	180±30	180±31
LDL‐C, mg/dL	150±27	151±27	149±27
HDL‐C, mg/dL	56.1±14.0	55.6±13.7	56.5±14.2
Triglycerides, mg/dL, median (IQR)	114 (85–153)	112 (84–146)	117 (87–158)
Apolipoprotein AI, mg/dL	145±26	144±26	146±26
Apolipoprotein B, mg/dL	122±22	122±21	121±22
RLP cholesterol, mg/dL, median (IQR)	4.8 (3.9–6.4)	4.7 (3.7–6.3)	4.9 (4.0–6.5)
sd‐LDL‐C, mg/dL, median (IQR)	32.7 (25.3–43.7)	32.7 (25.1–41.6)	32.8 (25.7–44.5)
NT‐proBNP, pg/mL, median (IQR)	118 (67–215)	116 (68–207)	119 (66–223)
hs‐CRP, mg/L, median (IQR)	0.57 (0.29–1.19)	0.63 (0.31–1.17)	0.55 (0.28–1.21)
Total bilirubin, mg/dL	0.58±0.23	0.57±0.23	0.59 (0.23)
eGFR, mL/min per 1.73 m^2^	62.3±16.6	62.7±16.9	62.0 (16.4)
Comorbid condition, n (%)			
Hypertension	929 (87.6)	438 (87.4)	491 (87.7)
Diabetes	308 (29.0)	138 (27.5)	170 (30.4)
Peripheral arterial disease	16 (1.5)	9 (1.8)	7 (1.3)
Number of risk factors†, n (%)			
1–2	905 (85.3)	427 (85.2)	478 (85.4)
≥3	156 (14.7)	74 (14.8)	82 (14.6)
Past history, n (%)			
Cerebral infarction	86 (8.1)	42 (8.4)	44 (7.9)
Fracture	32 (3.0)	21 (4.2)	11 (2.0)
Treatment of malignant tumors	15 (1.4)	6 (1.2)	9 (1.6)
Chronic obstructive pulmonary disease	9 (0.8)	4 (0.8)	5 (0.9)
Cerebral hemorrhage	9 (0.8)	4 (0.8)	5 (0.9)
Prior use of drugs for dyslipidemia			
Absent	807 (76.1)	383 (76.5)	424 (75.7)
Present	254 (23.9)	118 (23.6)	136 (24.3)

Values are mean±SD unless otherwise specified. eGFR indicates estimated glomerular filtration rate; HDL‐C, high‐density lipoprotein cholesterol; hs‐CRP, high‐sensitivity C‐reactive protein; LDL‐C, low‐density lipoprotein cholesterol; NT‐proBNP, N‐terminal pro‐B‐type natriuretic peptide; RLP, remnant‐like lipoprotein particle; and sd‐LDL‐C, small dense low‐density lipoprotein cholesterol.

* Missing values occurred to body mass index (n=6), waist circumference (n=11), blood pressure (n=4), sd‐LDL‐C (n=61), and hs‐CRP (n=31).

^†^ Risk factors included diabetes, hypertension, peripheral artery disease, low HDL‐C (<40 mg/dL), hypertriglyceridemia (≥ 150 mg/dL), current smoking, and history of cerebral infarction. All the patients had at least 1 condition.

**Table 2:** Cholesterol Absorption and Synthesis Markers, Oxycholesterols, and Phospholipids at Baseline

Variable	Both	Ezetimibe	Control
Absorption and synthesis markers*	(n=871)	(n=408)	(n=463)
Cholestanol	153 (123–186)	156 (127–189)	149 (119–182)
Desmosterol	58 (45–74)	58 (46–76)	57 (44–73)
Lathosterol	90 (64–126)	92 (64–128)	87 (64–125)
Campesterol	219 (156–302)	223 (161–296)	212 (154–304)
β‐Sitosterol	132 (97–183)	132 (99–183)	129 (93–183)
Campesterol/lathosterol ratio	2.37 (1.44–4.08)	2.21 (1.45–4.15)	2.51 (1.44–4.05)
Oxycholesterols†	(n=884)	(n=415)	(n=469)
Total oxycholesterols	131 (80–193)	133 (82–191)	130 (77–194)
7α‐Hydroxycholesterol	24 (14–37)	24 (15–37)	24 (13–37)
7β‐Hydroxycholesterol	31 (17–51)	31 (18–50)	31 (16–51)
β‐Epoxycholesterol	23 (14–37)	24 (14–38)	22 (14–34)
7‐Ketocholesterol	18 (9–30)	19 (9–31)	17 (8–29)
Phospholipids	(n=566)	(n=258)	(n=308)
PCOOH, nmol/L	388 (256–624)	406 (251–609)	375 (261–654)
Phosphatidylcholine, mmol/L	2.58 (2.17–3.07)	2.53 (2.14–2.96)	2.64 (2.19–3.13)
PCOOH/PC ratio	0.15 (0.10–0.23)	0.16 (0.10–0.23)	0.15 (0.11–0.23)

Values are medians (interquartile range). PCOOH indicates phosphatidylcholine hydroperoxide.

* ×100 μg per mg cholesterol.

^†^ ×100 ng per mg cholesterol.

### Cardiovascular Outcomes

The median observation period was 4.0 years (interquartile range, 2.7–4.9 years), and the number of primary outcomes was 64. Event‐specific outcomes were as follows: sudden cardiac death, 8; myocardial infarction, 9 (fatal, 3; nonfatal, 6); coronary revascularization, 14; cerebral infarction, 30 (fatal, 3; nonfatal, 27); and cerebral hemorrhage, 3 (fatal, 1; nonfatal, 2). In the KEEP study, ezetimibe treatment was associated with a statistically nonsignificant decrease in the risk of cardiovascular outcomes; the sex‐ and age‐adjusted HR was 0.65 (95% CI, 0.39–1.07; *P*=0.09), and the HR in model 2 was 0.62 (95% CI, 0.37–1.03; *P*=0.07).

The HRs of CVEs according to quartile categories of cholesterol absorption and synthesis markers at baseline are shown in Table 3. Results from the full adjustment for the covariates did not materially differ from those adjusted for sex, age, and treatment only. Higher campesterol levels at baseline were significantly associated with a decreased risk of CVEs, while other cholesterol absorption markers (cholestanol and β‐sitosterol) showed a similar but nonsignificant association. Lathosterol showed a nonsignificant increase in the risk of CVEs in the highest category. Higher campesterol‐to‐lathosterol ratios were associated with a lower risk of CVEs; the trend was statistically significant in model 1 (*P*=0.04) but not in model 2 (*P*=0.06). Furthermore, the effect of ezetimibe on risk of CVEs was examined in the low and high categories of the lathosterol, campesterol, and campesterol/lathosterol ratios (Table 4). The decreased risk of CVEs associated with ezetimibe treatment seemed to be more pronounced in the high category of lathosterol and in the low category of campesterol. However, the seemingly differential decreases in the risk were far from the statistical significance with respect to lathosterol (*P*=0.54) and campesterol (*P*=0.63).

**Table 3:** Hazard Ratios of the Cardiovascular Outcome According to Quartile Categories of Serum Cholesterol Absorption and Synthesis Markers (×100 μg per mg Cholesterol) at Baseline

Variable	Q1	Q2	Q3	Q4	Trend *P*
Cholestanol					
Range*	18–123	123–153	154–186	186–468	
Median	106	139	167	215	
Number†	21/218	13/218	11/218	11/217	
HR1 (95% CI)‡	1.00 (ref)	0.62 (0.31–1.25)	0.54 (0.26–1.12)	0.53 (0.25–1.11)	0.06
HR2 (95% CI)§	1.00 (ref)	0.66 (0.33–1.34)	0.56 (0.26–1.17)	0.49 (0.23–1.05)	0.05
Desmosterol					
Range*	4–45	45–58	58–74	75–343	
Median	38	51	64	90	
Number†	13/218	14/218	11/218	18/217	
HR1 (95% CI)‡	1.00 (ref)	1.03 (0.48–2.20)	0.86 (0.39–1.93)	1.38 (0.67–2.87)	0.46
HR2 (95% CI)§	1.00 (ref)	1.14 (0.53–2.45)	0.83 (0.37–1.89)	1.51 (0.71–3.23)	0.43
Lathosterol					
Range*	4–64	64–90	90–126	127–442	
Median	50	78	107	158	
Number†	12/218	11/218	13/218	20/217	
HR1 (95% CI)‡	1.00 (ref)	0.87 (0.38–1.99)	1.07 (0.48–2.37)	1.68 (0.81–3.49)	0.11
HR2 (95% CI)§	1.00 (ref)	0.88 (0.38–2.01)	1.08 (0.48–2.41)	1.61 (0.76–3.41)	0.15
Campesterol					
Range*	9–156	157–219	220–302‐	302–1573	
Median	121	185	256	383	
Number†	23/218	13/218	10/218	10/217	
HR1 (95% CI)‡	1.00 (ref)	0.59 (0.30–1.17)	0.44 (0.21–0.94)	0.44 (0.21–0.93)	0.01
HR2 (95% CI)§	1.00 (ref)	0.66 (0.33–1.34)	0.46 (0.22–0.98)	0.47 (0.22–1.01)	0.02
β‐Sitosterol					
Range*	6–97	97–132	132–183	183–865	
Median	78	115	153	226	
Number†	18/218	19/218	9/218	10/217	
HR1 (95% CI)‡	1.00 (ref)	1.15 (0.60–2.21)	0.50 (0.22–1.10)	0.59 (0.27–1.28)	0.05
HR2 (95% CI)§	1.00 (ref)	1.15 (0.59–2.23)	0.53 (0.23–1.20)	0.61 (0.28–1.36)	0.08
Campesterol/lathosterol ratio					
Range*	0.10–1.44	1.44–2.37	2.38–4.08	4.10–56.4	
Median	0.94	1.90	3.16	6.57	
Number†	18/218	17/218	14/218	7/217	
HR1 (95% CI)‡	1.00 (ref)	0.97 (0.50–1.88)	0.79 (0.39–1.60)	0.39 (0.16–0.94)	0.04
HR2 (95% CI)§	1.00 (ref)	1.01 (0.52–1.99)	0.86 (0.42–1.78)	0.41 (0.17–1.02)	0.06

HR indicates hazard ratio.

* Range. The minimum and maximum in the range were rounded to integer or 2 decimal places.

^†^ Number of event cases/patients.

^‡^ Adjusted for sex, age (<80, 80–84, and ≥85 years), and treatment group.

^§^ Adjusted for sex, age (<80, 80–84, and ≥85 years), treatment group, hypertension, diabetes, peripheral artery disease, history of cerebral infarction, prior use of drugs for dyslipidemia, baseline low‐density lipoprotein and high‐density lipoprotein cholesterol (quartile categories), and smoking (never, past, and current).

**Table 4:** Hazard Ratios of the Cardiovascular Outcome for the Ezetimibe Group Compared With Control Group in the Low and High Categories of Selected Serum Cholesterol Absorption and Synthesis Markers at Baseline

Variable	Group		Category*		Interaction
			Low	High	
Lathosterol					
	Diet	Number†	14/242	21/221	
		HR (95% CI)‡	1.00 (referent)	1.00 (referent)	*P*=0.54
	Ezetimibe	Number	9/194	12/214	
		HR (95% CI)	0.80 (0.34–1.89)	0.55 (0.26–1.13)	
Campesterol					
	Diet	Number	24/240	11/223	
		HR (95% CI)	1.00 (referent)	1.00 (referent)	*P*=0.63
	Ezetimibe	Number	12/196	9/212	
		HR (95% CI)	0.62 (0.30–1.28)	0.90 (0.35–2.30)	
Campesterol/lathosterol ratio					
	Diet	Number	22/221	13/242	
		HR (95% CI)	1.00 (referent)	1.00 (referent)	*P*=0.83
	Ezetimibe	Number	13/215	8/193	
		HR (95% CI)	0.61 (0.30–1.26)	0.76 (0.31–1.88)	

HR indicates hazard ratio.

* Two equal‐sized categories were determined in the whole subjects.

^†^ Number of event cases/patients.

^‡^ Adjusted for sex, age (<80, 80–84, and 85+ years), hypertension, diabetes, peripheral artery disease, history of cerebral infarction, quartile categories of baseline low‐density lipoprotein and high‐density lipoprotein cholesterol, prior use of drugs for dyslipidemia, and smoking (never, past, and current).

The associations between lipid parameters and other laboratory measurements at baseline and risk of CVEs are summarized in Tables S1 and S2. Again, the results did not differ in model 1 and model 2. Higher levels of NT‐proBNP were significantly associated with risk of CVEs. The positive association with NT‐proBNP did not change measurably after additional adjustment for estimated glomerular filtration rate in model 2; the adjusted HR from the first to fourth quartile categories of NT‐proBNP were 1.00 (reference), 2.51 (95% CI, 1.07–5.89), 1.79 (95% CI, 0.73–4.39), and 3.26 (95% CI, 1.40–7.63), respectively (trend *P*=0.02). Among the oxycholesterols, 7β‐hydroxycholesterol showed a tendency toward a positive association with the risk of CVEs (trend *P*=0.05). The PCOOH/phosphatidylcholine ratio also showed a statistically nonsignificant positive association with risk of CVEs (trend *P*=0.08).

### Changes From Baseline in Laboratory Measurements

Of the 1061 study patients, 55 did not have blood sampling at 24 weeks, and 11 patients exited the study before the 24‐week visit (6 with the primary outcome and 5 without the primary outcome). Analysis of the changes from baseline at 24 weeks was performed in 995 patients. Further attrition in the number of patients was observed in the analyses of cholesterol absorption and synthesis markers, oxycholesterol, and phospholipids.

Substantial decreases were noted in total cholesterol, non–HDL‐C, LDL‐C, apolipoprotein B, remnant‐like lipoprotein particle cholesterol, and small dense LDL‐C in the ezetimibe group, and increases in HDL‐C and apolipoprotein AI were near significantly or significantly greater in the ezetimibe group than in the control group (Table 5). There were no distinct between‐group differences in the changes in NT‐proBNP or hsCRP levels.

**Table 5:** Percent Changes From Baseline in Lipid‐Related Parameters and Other Laboratory Measurements at 24 Weeks in the Ezetimibe and Control Group

Variable	Ezetimibe (n=473)	Control (n=522)	*P* value*
Total cholesterol, mg/dL	−10.8 (−11.8 to −9.7)	−1.7 (−2.7 to −0.7)	<10^−31^
Non–HDL‐C, mg/dL	−14.4 (−15.7 to −13.1)	−2.0 (−3.2 to −0.7)	<10^−38^
LDL‐C, mg/dL	−14.8 (−16.3 to −13.4)	−1.9 (−3.2 to −0.5)	<10^−34^
HDL‐C, mg/dL	2.5 (1.2 to 3.8)	0.8 (−0.5 to 2.0)	0.06
Triglycerides, mg/dL	−3.0 (−5.7 to −0.1)	−2.0 (−4.7 to 0.7)	0.65
Apolipoprotein AI, mg/dL	2.6 (1.6 to 3.6)	0.5 (−0.4 to 1.5)	0.004
Apolipoprotein B, mg/dL	−12.7 (−14.0 to −11.5)	−1.7 (−2.9 to −0.5)	<10^−32^
RLP cholesterol, mg/dL	−17.0 (−19.8 to −14.2)	−4.9 (−7.9 to −1.8)	<10^−7^
sd‐LDL‐C, mg/dL†	−15.4 (−17.7 to −13.1)	−1.2 (−3.8 to 1.4)	<10^−14^
NT‐proBNP, pg/mL	1.0 (−3.4 to 5.7)	3.4 (−1.0 to 7.9)	0.48
hs‐CRP, mg/L‡	1.5 (−8.1 to 12.0)	10.2 (0.3 to 21.2)	0.24
Total bilirubin, mg/dL	2.0 (−0.7 to 4.6)	6.2 (3.7 to 8.7)	0.02
eGFR, mL/min per 1.73 m^2^	0.6 (−0.5 to 1.8)	−0.5 (−1.6 to 0.6)	0.15

Values are adjusted mean percent changes (95% confidence intervals) derived from analysis of covariance controlling for the baseline value. eGFR indicates estimated glomerular filtration rate; HDL‐C, high‐density lipoprotein cholesterol; hs‐CRP, high‐sensitivity C‐reactive protein; IQR, interquartile range; NT‐proBNP, N‐terminal pro‐B‐type natriuretic peptide; RLP, remnant‐like lipoprotein particle; and sd‐LDL‐C, small dense low‐density lipoprotein cholesterol.

* *P* value for the between‐group difference.

^†^ Subjects numbered 440 in ezetimibe group and 485 in control group.

^‡^ Subjects numbered 464 in ezetimibe group and 505 in control group.

In the ezetimibe group, cholesterol absorption markers and the campesterol/lathosterol ratio decreased substantially, whereas cholesterol synthesis markers increased notably. No such changes were observed in the control group. Neither oxycholesterols nor phospholipids showed measurable between‐group differences in changes from baseline (Table 6). Median changes in all analyzed molecules of cholesterol absorption/synthesis markers and oxycholesterols are depicted in Figures S3 and S4.

**Table 6:** Percentage Changes From Baseline in Cholesterol Absorption and Synthesis Markers and Oxycholesterols at 24 Weeks in the Ezetimibe and Control Group

Variable	Ezetimibe	Control	*P* value*
Absorption and synthesis markers†	(n=393)	(n=451)	
Cholestanol	−4.4 (−6.7 to −2.2)	1.2 (−1.0 to 3.4)	<10^−3^
Desmosterol	25.9 (21.2 to 30.7)	−0.6 (−4.0 to 3.0)	<10^−17^
Lathosterol	42.0 (36.0 to 48.3)	1.0 (−2.9 to 5.2)	<10^−26^
Campesterol	−45.5 (−47.8 to −43.1)	−0.8 (−4.7 to 3.3)	<10^−71^
β‐Sitosterol	−33.1 (−35.5 to −30.6)	−2.5 (−5.8 to 0.9)	<10^−42^
Campesterol/lathosterol ratio	−61.6 (−64.1 to −58.8)	−1.9 (−8.1 to 4.6)	<10^−68^
Oxycholesterols‡	(n=405)	(n=456)	
Total oxycholesterols	−6.5 (−11.7 to −1.1)	−9.6 (−14.3 to −4.6)	0.41
7α‐Hydroxycholesterol	−9.9 (−16.5 to −2.8)	−12.7 (−18.7 to −6.2)	0.56
7β‐Hydroxycholesterol	−16.3 (−23.0 to −9.0)	−19.6 (−25.7 to −13.0)	0.49
β‐Epoxycholesterol	−0.8 (−7.1 to 5.8)	−3.7 (−9.4 to 2.4)	0.53
7‐Ketocholesterol	−7.0 (−14.9 to 1.7)	−9.3 (−16.6 to −1.4)	0.69
Phospholipids	(n=230)	(n=266)	
PCOOH, nmol/L	−18.0 (−25.4 to −9.8)	−17.8 (−24.8 to −10.2)	0.98
Phosphatidylcholine, mmol/L	−16.7 (−19.9 to −13.3)	−8.6 (−11.9 to −5.1)	<10^−3^
PCOOH/phosphatidylcholine ratio	−1.0 (−9.3 to 8.2)	−10.2 (−17.3 to −2.5)	0.11

Values are adjusted mean percent changes (95% confidence intervals) derived from analysis of covariance controlling for the baseline value. PCOOH indicates phosphatidylcholine hydroperoxide.

* *P* value for the between‐group difference.

^†^ ×100 μg per mg cholesterol.

^‡^ ×100 ng per mg cholesterol.

### Causal Mediation Analysis on Changes in Cholesterol Absorption Markers

A causal mediation analysis indicated no measurable mediation attributable to the changes in cholesterol absorption markers in the reduced risk of CVEs associated with ezetimibe treatment (Table 7). The overall proportion explained by mediating effect (pure indirect effect and mediated interaction combined) was estimated to be 16% (95% CI, −16 to 48) for cholestanol, 23% (95% CI, −75 to 121) for campesterol, 5% (95% CI, −72 to 81) for sitosterol E, and 26% (95% CI, −83 to 136) for campesterol/lathosterol ratio. These proportions per se were apparently large except for sitosterol E, but the 95% CIs were fairly wide including zero. On the other hand, the controlled direct effect, that is, the ezetimibe effect through pathways independent of a cholesterol absorption marker, accounted for the majority of the reduced risk of cardiovascular outcome associated with ezetimibe treatment in each analysis.

**Table 7:** Four‐Way Decomposition Causal Mediation Analyses on Cholesterol Absorption Markers (Changes From Baseline at 24 Weeks) in the Association Between Ezetimibe Treatment and Cardiovascular Outcome

Mediator/component	Excess relative risk	(95% CI)	Proportion attributable (%)	(95% CI)
Cholestanol				
Controlled direct effect	−0.33	(−0.73 to 0.07)	98	(77 to 118)
Reference interaction	0.04	(−0.06 to 0.15)	−13	(−51 to 24)
Mediated interaction	−0.03	(−0.13 to 0.06)	10	(−22 to 43)
Pure indirect effect	−0.02	(−0.10 to 0.06)	6	(−19 to 30)
Total effect	−0.34	(−0.73 to 0.05)	100	
Campesterol				
Controlled direct effect	−0.34	(−0.83 to 0.14)	109	(33 to 186)
Reference interaction	0.10	(−0.14 to 0.34)	−32	(−118 to 54)
Mediated interaction	−0.23	(−0.84 to 0.37)	74	(−137 to 284)
Pure indirect effect	0.16	(−0.39 to 0.71)	−51	(−234 to 132)
Total effect	−0.31	(−0.72 to 0.09)	100	
β‐Sitosterol				
Controlled direct effect	−0.24	(−0.71 to 0.23)	78	(−10 to 166)
Reference interaction	−0.05	(−0.30 to 0.19)	18	(−65 to 100)
Mediated interaction	0.10	(−0.31 to 0.51)	−32	(−173 to 109)
Pure indirect effect	−0.11	(−0.47 to 0.24)	37	(−87 to 161)
Total effect	−0.31	(−0.71 to 0.10)	100	
Campesterol/lathosterol ratio				
Controlled direct effect	−0.31	(−0.82 to 0.20)	95	(7 to 184)
Reference interaction	0.07	(−0.22 to 0.36)	−21	(−113 to 71)
Mediated interaction	−0.14	(−0.77 to 0.49)	44	(−155 to 244)
Pure indirect effect	0.06	(−0.50 to 0.62)	−18	(−190 to 154)
Total effect	−0.33	(−0.72 to 0.07)	100	

Included as covariates were sex, age (<80, 80–84, and ≥85 years), baseline value of the parameter of interest (quartile categories), hypertension, diabetes, peripheral artery disease, history of cerebral infarction, prior use of drugs for dyslipidemia, baseline low‐density lipoprotein cholesterol and high‐density lipoprotein cholesterol(quartile categories), and smoking (never, past, and current). Mediators were the changes in the values transformed to the natural‐logarithm scale.

### Laboratory Markers for Safety

Laboratory adverse events were investigated among the 995 patients with measurements at 24 weeks (n=473 for ezetimibe and n=522 for control). The number of patients with the Common Terminology Criteria Adverse Events–defined adverse events (grade 1+) were 9 for AST (ezetimibe, 5; control, 4; chi‐square test *P*=0.63), 18 for ALT (ezetimibe, 7; control, 11; *P*=0.46), 40 for creatinine phosphokinase (ezetimibe, 21; control, 19; *P*=0.52), and 56 for creatinine (ezetimibe, 18; control, 38; *P*=0.02). The adverse events of grade 2+ were rare: null for AST, 1 for ALT (ezetimibe group), 2 for creatinine phosphokinase (ezetimibe group), and 1 for creatinine (control group). None had the adverse events of grade ≥3.

## Discussion

The findings of the KEEP study suggest that baseline campesterol level may be a useful marker for predicting risk of CVEs in older old patients with hypercholesterolemia aged ≥75 years. The effect of ezetimibe on risk of CVEs was not dependent on the campesterol level. Also, the usefulness of other cholesterol synthesis or absorption markers, such as sitosterol, cholestanol, and lathosterol, for predicting CVEs was not clear. The study found that NT‐proBNP, but not hs‐CRP, was significantly associated with risk of CVEs. The present study failed to substantiate the mediating effects of the changes in cholesterol absorption markers in the reduced risk of CVEs associated with ezetimibe treatment.

While some studies have shown a positive association between campesterol or absorption marker levels and cardiovascular risk,^28^, ^29^, ^30^ others have reported conflicting results.^31^, ^32^, ^33^ A study demonstrated that plasma campesterol was positively associated with carotid plaques in asymptomatic subjects.^28^ Another study found that the combined reduction of campesterol and lathosterol predicts future cardiac events in patients with early‐stage nonischemic dilated cardiomyopathy.^29^ In patients with severe aortic stenosis scheduled for elective aortic valve replacement, the absolute values for campesterol were increased in the tissues of patients with documented coronary artery disease, as well as campesterol oxides in the aortic valve cusps.^30^ In the Framingham Offspring Study, campesterol, sitosterol, and cholestanol were associated with coronary artery disease, while lathosterol was negatively related to the coronary disease.^31^ However, contrasting results have been reported regarding the relationship between cholesterol absorption and synthesis markers and CVEs. Campesterol and sitosterol were unrelated to coronary artery disease in the EPIC‐Norfolk (European Prospective Investigation Into Cancer–Norfolk) cohort study.^32^ Furthermore, the Spanish EPIC cohort study found that higher levels of plasma sitosterol and campesterol were associated with a decreased risk of coronary heart disease.^33^ The authors attributed this protective association to the high intake of plant foods in individuals with elevated levels of sitosterol and campesterol.

The KEEP study revealed fewer CVEs in older old patients with elevated campesterol levels at the baseline who underwent treatment for hypercholesterolemia. Initially, this might seem contradictory, as most of the previous studies indicated that the high campesterol group had a higher risk of CVEs. However, the findings of the KEEP study suggested that the treatment for hypercholesterolemia was more successful in this population with elevated campesterol levels at the baseline, resulting in a reduced number of CVEs. It may be interesting to examine whether the decrease in LDL‐C by treatment differs according to campesterol levels at baseline. There was no association between baseline campesterol and the magnitude of LDL reduction in either ezetimibe or control group. With adjustment for baseline LDL‐C by analysis of covariance, the decreases in LDL‐C in the first to fourth quartile categories of baseline campesterol levels were 21.7, 27.1, 25.5, and 22.7 mg/dL, respectively, in the ezetimibe group (*P*=0.27), and the corresponding values in the control group were 4.1, 4.8, 4.7, and 3.0 mg/dL, respectively (*P*=0.95). The observed inverse association between baseline campesterol and risk of CVEs does not seem to be linked to the reduction in LDL‐C.

It is well known that cholesterol metabolism changes with aging. In general, serum cholesterol levels are elevated in the elderly population. In a study of adults aged 38 to 79 years conducted in Italy, however, cholesterol synthesis as determined by lathosterol/cholesterol ratio was shown to decrease progressively with aging.^22^ The apparent paradoxical phenomena seem to be due to a decreased metabolism of cholesterol with aging. Cholesterol absorption markers did not clearly decrease with aging.^22^ In this regard, the therapeutic strategy for lowering cholesterol necessarily differs between midlife adults and elderly adults. The KEEP study demonstrated that ezetimibe was associated with a moderate reduction in the incidence of CVEs, although the statistical significance was limited due to the small number of subjects in the KEEP study. The HR of CVEs associated with ezetimibe in the KEEP study was similar to that observed in the EWTOPIA 75 study, indicating the findings are consistent with those observed in the EWTOPIA 75 study.^12^

The implications of our results for clinical practice require careful consideration. While baseline campesterol levels were found to be predictive of CVEs, the therapeutic efficacy of ezetimibe did not differ by campesterol levels with respect to LDL‐C reduction. Furthermore, the present study did not support an idea that ezetimibe effect on the risk of CVEs might be differential with baseline campesterol levels. Further studies are needed as to clinical use of cholesterol absorption/synthesis in relation to ezetimibe treatment.

The results of the KEEP study should be interpreted in the context of its limitations. The study was conducted among patients aged >75 years in a Japanese population, which may limit the generalizability of the findings to younger patients and other races. Previous studies, such as the MEGA (Management of Elevated Cholesterol in the Primary Prevention Group of Adult Japanese) study^34^ and EWTOPIA 75 study^12^ demonstrated that the effects of lipid‐lowering on primary prevention of CVEs were greater in the Japanese than in other races. Therefore, these results should be interpreted cautiously, considering the need for further research to confirm the findings and determine their generalizability to other races. Moreover, the Japanese diet is often perceived as healthy due to its distinct polyunsaturated fatty acid content and high consumption of soy foods. The KEEP study did not investigate the participants' diets, thereby causing a limitation in extending the present findings to non‐Japanese populations. Additionally, the KEEP study faced limitations similar to the EWTOPIA75 study, such as an open‐label design and early termination of the study. The independent data monitoring committee of the EWTOPIA 75 study recommended terminating the study earlier to prevent prolonged follow‐up and issues with competing risks and loss to follow‐up.^12^ The exclusion criteria for eligible patients included a vague “inappropriateness for enrollment as judged by the attending physician,” which had the potential to affect the clarity of the participant pool. This criterion was included to avoid an unanticipated situation in which enrollment could pose potential risks to participants. While such discretion‐based exclusions are common in Japanese clinical trials^35^, ^36^ this criterion needs to be reconsidered. In the EWTOPIA 75 study,^12^ the ezetimibe effect on CVEs was slightly, nonsignificantly greater in those with prior use of drugs for dyslipidemia treatment than in those without. Stratification with the prior use of lipid‐lowering drugs may provide valuable information on the association between campesterol and CVEs, but the number of those with such a history was not large enough to do a stratified analysis; those with the prior use of lipid‐lowering drugs and statins accounted for 24% (n=254) and 20% (n=220), respectively.

The follow‐up of 24 weeks may not have been long enough to evaluate the changes in cholesterol absorption/synthesis markers and other laboratory parameters during the treatment. The change based on measurements at only 2 points in time (baseline and 24 weeks) may not have represented the true individual's change from baseline during the treatment. In addition to the limited size of the study, these points may be an explanation for having failed to find a mediating effect of the changes in cholesterol absorption markers. Finally, it may be argued that multiple comparisons should have been taken into consideration because many associations with multiple parameters were tested statistically. Cholesterol absorption and synthesis markers were of primary interest, but the statistically significant or near significant findings on other parameters such as NT‐proBNP and a specific oxycholesterol need to be corroborated in further studies.

In conclusion, the KEEP study indicated that the higher levels of campesterol not on lipid‐lowering drugs were associated with a lower incidence of CVEs in hypercholesterolemic older old individuals aged ≥75 years who were subsequently treated with diet or ezetimibe. These findings provide a rationale for treating patients with hypercholesterolemia with ezetimibe for the prevention of CVEs when pharmacotherapy is indicated. Further research is needed to confirm these findings in other populations and to investigate the mechanisms underlying the association between cholesterol absorption markers and CVEs.

## Sources of Funding

The company distributing the trial drug (Bayer Yakuhin, Ltd.) and the Okinaka Memorial Institute for Medical Research provided financial support for the projects but were not involved in the design, analysis, data interpretation, or manuscript preparation.

## Disclosures

Dr Ouchi and Dr Kuwabara received support from the Okinaka Memorial Institute for Medical Research. The remaining authors have no disclosures to report.

## Supporting information

Tables S1–S2
Figures S1–S4
