# Supplementary material for: Higher Cholesterol Absorption Marker at Baseline Predicts Fewer Cardiovascular Events in Elderly Patients Receiving Hypercholesterolemia Treatment: The KEEP Study
Source: J Am Heart Assoc. 2024 Jan 19;13(3):e031865. doi: 10.1161/JAHA.123.031865 (PMC11056156; doi:10.1161/JAHA.123.031865)
Supplement: Supplementary file 1 — Tables S1–S2 Figures S1–S4 [file JAH3-13-e031865-s001.pdf]

Supplemental Material

Table S1. Hazard ratios of the cardiovascular outcome according to quartile categories of lipid parameters and other laboratory measurements at baseline.

Variable	Q1	Q2	Q3	Q4	Trend *p*
LDL cholesterol (mg/dL)					
Number*	19/269	13/268	14/265	18/259	
HR1 (95% CI)†	1.00 (ref)	0.64 (0.32–1.30)	0.74 (0.37–1.49)	0.97 (0.51–1.85)	0.98
HR2 (95% CI)‡	1.00 (ref)	0.65 (0.32–1.32)	0.83 (0.41–1.68)	1.13 (0.58–2.20)	0.63
HDL cholesterol (mg/dL)					
Number	21/293	17/272	14/247	12/249	
HR1 (95% CI)†	1.00 (ref)	0.84 (0.44–1.59)	0.79 (0.40–1.55)	0.70 (0.34–1.44)	0.32
HR2 (95% CI)‡	1.00 (ref)	0.86 (0.45–1.65)	0.85 (0.42–1.72)	0.76 (0.37–1.58)	0.47
Apolipoprotein AI (mg/dL)					
Number	21/278	20/263	11/267	12/253	
HR1 (95% CI)†	1.00 (ref)	0.99 (0.54–1.83)	0.54 (0.26–1.12)	0.67 (0.33–1.38)	0.11
HR2 (95% CI)‡	1.00 (ref)	1.15 (0.61–2.17)	0.58 (0.27–1.24)	0.75 (0.36–1.57)	0.19
Apolipoprotein B (mg/dL)					
Number	21/279	9/264	11/261	23/257	
HR1 (95% CI)†	1.00 (ref)	0.44 (0.20–0.97)	0.52 (0.25–1.09)	1.20 (0.66–2.17)	0.51
HR2 (95% CI)‡	1.00 (ref)	0.45 (0.20–0.98)	0.54 (0.25–1.14)	1.29 (0.69–2.42)	0.41
RLP cholesterol (mg/dL)					
Number	14/283	18/255	15/267	17/256	
HR1 (95% CI)†	1.00 (ref)	1.51 (0.75–3.06)	1.16 (0.56–2.42)	1.50 (0.74–3.05)	0.41
HR2 (95% CI)‡	1.00 (ref)	1.41 (0.69–2.92)	1.07 (0.50–2.30)	1.31 (0.61–2.78)	0.68
sd-LDL cholesterol (mg/dL)					
Number	15/251	11/250	14/249	19/250	
HR1 (95% CI)†	1.00 (ref)	0.71 (0.33–1.56)	0.95 (0.45–1.97)	1.25 (0.63–2.49)	0.39
HR2 (95% CI)‡		0.87 (0.37–2.06)	1.12 (0.46–2.69)	1.37 (0.56–3.33)	0.38
NT-pro BNP (pg/mL)					
Events	8/269	18/263	14/268	24/261	
HR1 (95% CI)†	1.00 (ref)	2.66 (1.15–6.17)	2.02 (0.83–4.90)	3.70 (1.60–8.57)	0.006
HR2 (95% CI)‡	1.00 (ref)	2.60 (1.11–6.07)	1.87 (0.77–4.58)	3.41 (1.47–7.92)	0.01
hs-CRP (mg/L)					
Number	15/258	14/259	13/257	20/256	
HR1 (95% CI)†	1.00 (ref)	0.95 (0.46–1.97)	0.88 (0.42–1.86)	1.34 (0.69–2.63)	0.42
HR2 (95% CI)‡	1.00 (ref)	0.90 (0.43–1.88)	0.81 (0.38–1.73)	1.19 (0.59–2.37)	0.66
Total bilirubin (mg/dL)					
Number	24/309	12/237	17/330	11/185	
HR1 (95% CI)†	1.00 (ref)	0.60 (0.30–1.21)	0.60 (0.32–1.13)	0.68 (0.33–1.42)	0.18
HR2 (95% CI)‡	1.00 (ref)	0.67 (0.33–1.37)	0.68 (0.36–1.29)	0.77 (0.36–1.64)	0.36
eGFR (mL/min/1.73 m^2)					
Number	20/286	17/266	17/251	10/258	
HR1 (95% CI)†	1.00 (ref)	0.89 (0.47–1.71)	0.96 (0.50–1.84)	0.57 (0.27–1.23)	0.22
HR2 (95% CI)‡	1.00 (ref)	0.86 (0.45–1.67)	1.14 (0.59–2.23)	0.61 (0.28–1.33)	0.39

CI, confidence interval; eGFR, estimated glomerular filtration rate; HR, hazard ratio, hs-CRP, high-sensitivity C-reactive protein; NT-pro BNP, N-terminal pro brain natriuretic peptide; RLP, remnant lipoprotein; sd-LDL, small-dense LDL.

*Number of event cases/patients.

†Adjusted for sex, age (<80, 80–84, and ≥85 years), and treatment group.

‡Adjusted for sex, age (<80, 80–84, and ≥85 years), treatment group, hypertension, diabetes mellitus, peripheral artery disease, history of cerebral infarction, quartile categories of baseline LDL (except for LDL cholesterol and apolipoprotein B) and HDL cholesterol (except for HDL cholesterol and apolipoprotein A), prior use of drugs for dyslipidemia, and smoking (never, past, and current).

Table S2. Hazard ratios of the cardiovascular outcome according to quartile categories of serum oxycholesterols (×100 ng per mg cholesterol) and phospholipid parameters at baseline.

Variable	Q1	Q2	Q3	Q4	Trend *p*
Total oxycholesterols					
Median	57.9	104.9	158.5	246.0	
Number*	13/221	13/221	9/221	21/221	
HR1 (95% CI)†	1.00 (ref)	0.86 (0.40–1.85)	0.57 (0.24–1.35)	1.31 (0.66–2.64)	0.50
HR2 (95% CI)‡	1.00 (ref)	0.94 (0.43–2.06)	0.61 (0.26–1.44)	1.39 (0.69–2.81)	0.44
7α-Hydroxycholesterol					
Median	8.1	18.5	29.6	47.6	
Number*	12/221	9/221	18/221	17/221	
HR1 (95% CI)†	1.00 (ref)	0.67 (0.28–1.60)	1.22 (0.58–2.54)	1.17 (0.55–2.46)	0.38
HR2 (95% CI)‡	1.00 (ref)	0.64 (0.27–1.55)	1.36 (0.63–2.91)	1.18 (0.55–2.52)	0.32
7β-Hydroxycholesterol					
Median	10.5	24.1	39.6	65.3	
Number*	9/221	10/221	17/221	20/221	
HR1 (95% CI)†	1.00 (ref)	0.89 (0.36–2.21)	1.51 (0.67–3.40)	1.76 (0.80–3.89)	0.06
HR2 (95% CI)‡	1.00 (ref)	0.83 (0.33–2.06)	1.65 (0.72–3.76)	1.76 (0.79–3.93)	0.05
β-Epoxycholesterol					
Median	10.2	18.3	29.2	48.2	
Number*	14/221	10/221	18/221	14/221	
HR1 (95% CI)†	1.00 (ref)	0.69 (0.30–1.55)	1.16 (0.58–2.35)	0.89 (0.42–1.88)	0.89
HR2 (95% CI)‡	1.00 (ref)	0.87 (0.38–2.00)	1.30 (0.64–2.66)	0.98 (0.45–2.12)	0.77
7-Ketocholesterol					
Median	5.0	13.5	23.4	42.6	
Number*	14/221	10/221	17/221	15/221	
HR1 (95% CI)†	1.00 (ref)	0.62 (0.27–1.39)	1.04 (0.51–2.11)	0.87 (0.42–1.81)	0.95
HR2 (95% CI)‡	1.00 (ref)	0.59 (0.26–1.35)	1.04 (0.51–2.13)	0.89 (0.42–1.86)	0.89
PCOOH (nM)					
Median	213	316	508	878+	
Number*	6/143	5/140	8/142	13/141	
HR1 (95% CI)†	1.00 (ref)	0.84 (0.25–2.77)	1.11 (0.38–3.21)	1.74 (0.65–4.64)	0.18
HR2 (95% CI)‡	1.00 (ref)	0.89 (0.26–2.96)	1.02 (0.35–3.04)	1.86 (0.68–5.07)	0.17
PC (mM)					
Median	1.86	2.37	2.79	3.50+	
Number*	6/143	11/140	9/144	6/139	
HR1 (95% CI)†	1.00 (ref)	1.64 (0.61–4.45)	1.14 (0.40–3.24)	0.70 (0.22–2.23)	0.37
HR2 (95% CI)‡	1.00 (ref)	1.64 (0.59–4.54)	1.11 (0.37–3.36)	0.67 (0.20–2.27)	0.34
PCOOH/PC ratio					
Median	0.08	0.13	0.19	0.32+	
Number*	6/151	5/138	9/140	12/137	
HR1 (95% CI)†	1.00 (ref)	1.01 (0.31–3.32)	1.61 (0.57–4.55)	2.13 (0.79–5.73)	0.09
HR2 (95% CI)‡	1.00 (ref)	0.97 (0.29–3.23)	1.41 (0.49–4.03)	2.21 (0.81–5.99)	0.08

CI: confidence interval; HR: hazard ratio; PCOOH: phosphatidylcholine hydroperoxide, PC: phosphatidylcholine.

*Number of event cases/patients.

†Adjusted for sex, age (<80, 80–84, and ≥85 years), and treatment group.

‡Adjusted for sex, age (<80, 80–84, and ≥85 years), treatment group, hypertension, diabetes mellitus, peripheral artery disease, history of cerebral infarction, quartile categories of baseline LDL and HDL cholesterol, prior use of drugs for dyslipidemia, and smoking (never, past, and current).

Figure S1. Distribution of analyzed cholesterol absorption and synthesis markers.

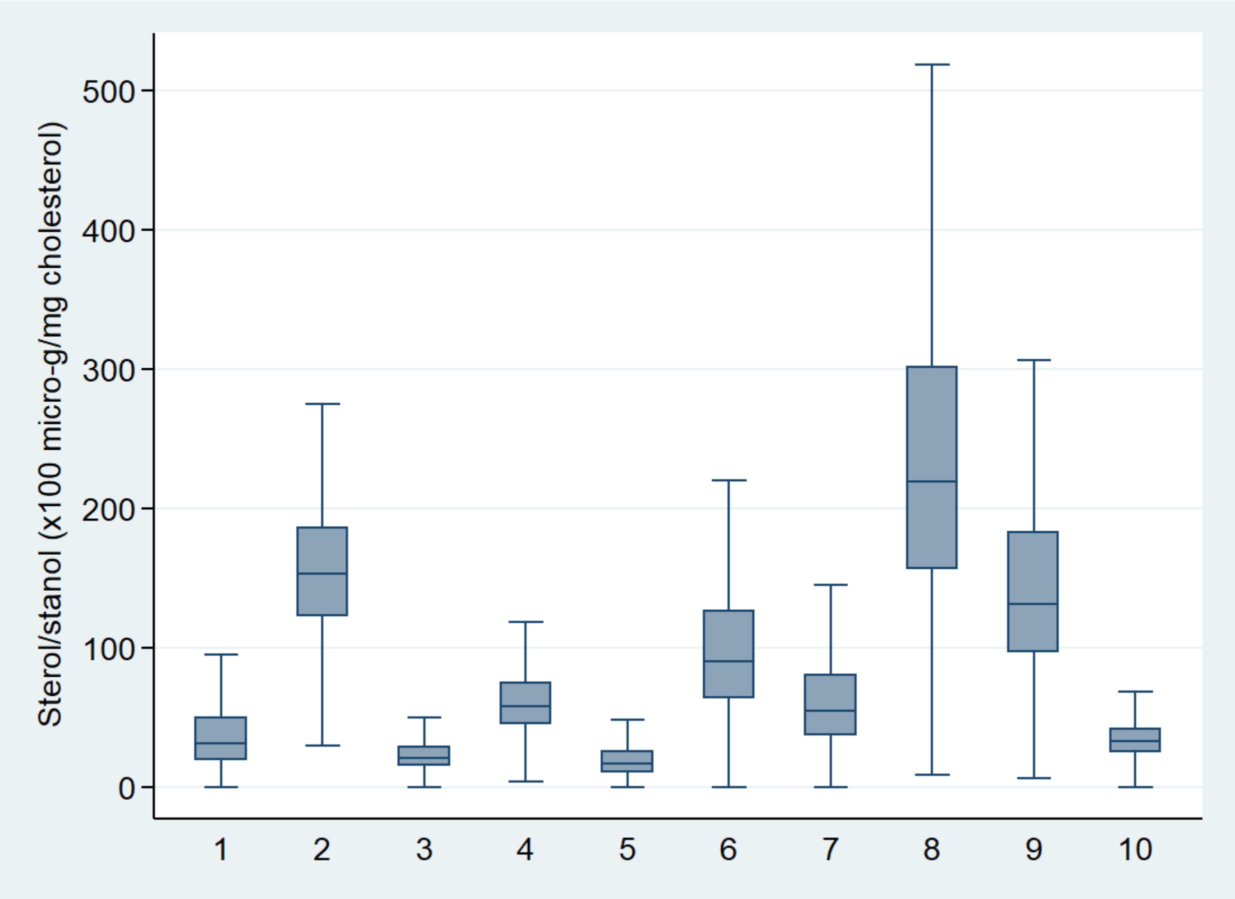

The numbers at the horizontal axis represent individual molecules:

1. Squalene*, 2. cholestanol, 3. 8-dehydrocholesterol, 4. Desmosterol*, 5. 7-dehydrocholesterol, 6. Lathosterol*, 7. unknown molecule, 8. campesterol, 9. β-sitosterol, 10. β-sitostanol.

*Asterisk indicates cholesterol synthesis marker.

Figure S2. Distribution of analyzed oxycholesterols.

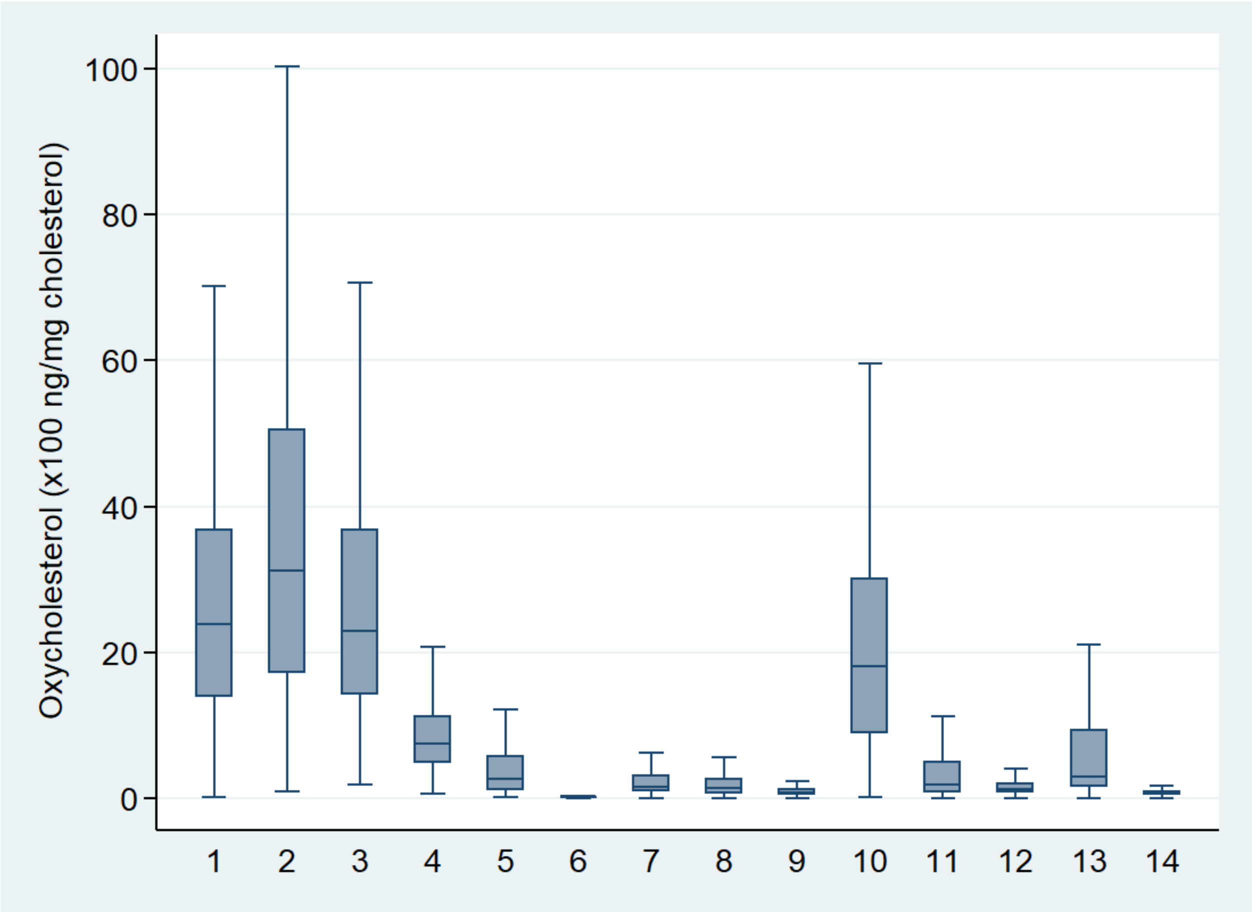

The numbers at the horizontal axis represent individual oxycholesterols:

1. 7α-hydroxycholesterol, 2. 7β-hydroxycholesterol, 3. β-epoxycholesterol, 4. α-epoxycholesterol , 5. 4β-hydroxycholesterol, 6. 22(R)-hydroxycholesterol, 7. β-cholestantriol, 8. 6-ketocholestanol, 9. α-cholestantriol, 10. 7-ketocholesterol, 11. 24(S)-hydroxycholesterol, 12. 25-hydroxycholesterol, 13. 27-hydroxycholesterol, 14. 5α-hydroxy,6-ketocholestanol.

Figure S3. Median changes from baseline in the serum levels of cholesterol absorption and synthesis markers at 24 weeks in the ezetimibe group and the control group.

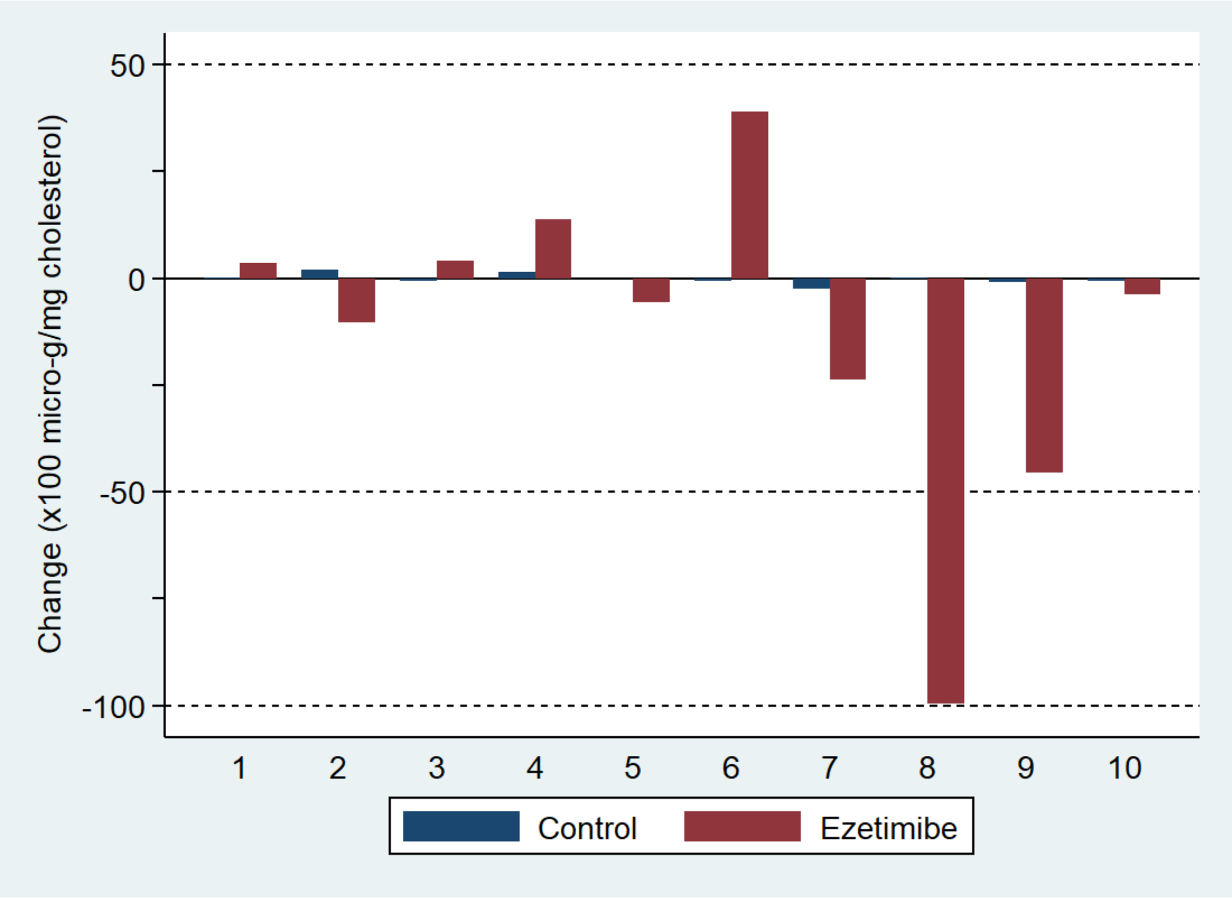

The numbers at the horizontal axis represent individual molecules:

1. Squalene*, 2. cholestanol, 3. 8-dehydrocholesterol, 4. Desmosterol*, 5. 7-dehydrocholesterol, 6. Lathosterol*, 7. unknown molecule, 8. campesterol, 9. β-sitosterol, 10. β-sitostanol.

*Asterisk indicates synthesis marker.

Except for squalene, the between-group difference was statistically significant for cholestanol ($P < 10^{-7}$), 8-dehydrocholesterol ($P < 10^{-7}$), desmosterol ($P < 10^{-14}$), 7-dehydrocholesterol ($P < 10^{-11}$), lathosterol ($P < 10^{-28}$), unknown molecule ($P < 10^{-31}$), campesterol ($P < 10^{-54}$), β-sitosterol ($P < 10^{-38}$), and β-sitostanol ($P < 10^{-4}$).

Figure S4. Median changes from baseline in the serum levels of oxycholesterols at 24 weeks in the ezetimibe group and the control group.

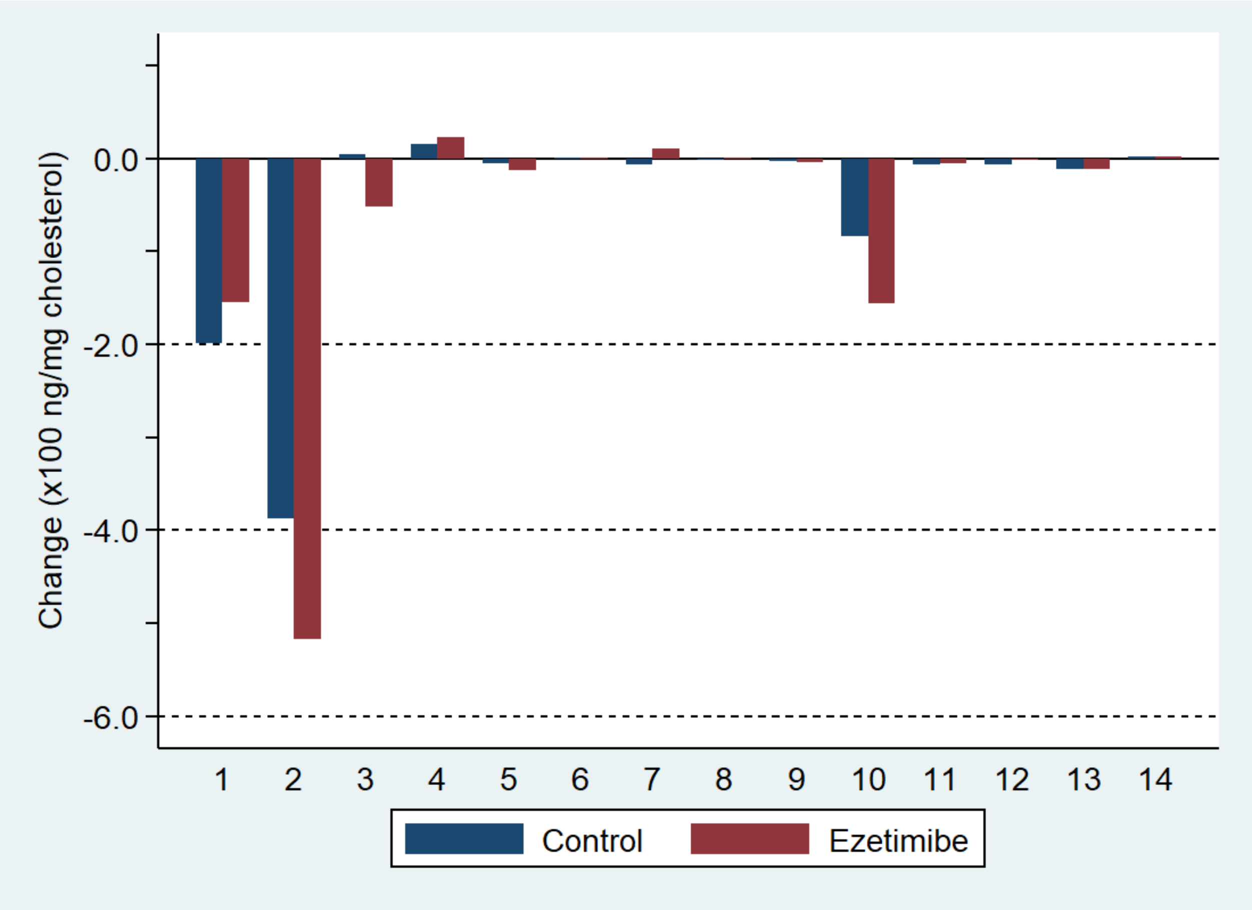

The numbers at the horizontal axis represent individual oxycholesterols:

1. 7α-hydroxycholesterol, 2. 7β-hydroxycholesterol, 3. β-epoxycholesterol, 4. α-epoxycholesterol , 5. 4β-hydroxycholesterol, 6. 22(R)-hydroxycholesterol, 7. β-cholestantriol, 8. 6-ketocholestanol, 9. α-cholestantriol, 10. 7-ketocholesterol, 11. 24(S)-hydroxycholesterol, 12. 25-hydroxycholesterol, 13. 27-hydroxycholesterol, 14. 5α-hydroxy,6-ketocholestanol.

The between-group difference was statistically significant only for β-cholestantriol ($P = 0.004$).
